# The Rolling Transition in a Granular Flow along a Rotating Wall

**DOI:** 10.3390/ma4112003

**Published:** 2011-11-11

**Authors:** Francois Rioual, Aurélie Le Quiniou, Yuri Lapusta

**Affiliations:** 1 Cemagref Grenoble, 2 rue de la Papeterie, St Martin d’Hères BP 76 38402, France; 2 Cemagref Clermont-Ferrand, Domaine des Palaquins, Montoldre BP 03150, France; E-Mail: aurelie.lequiniou@aol.com; 3 Institut Francais de Mécanique Avancée, Campus de Clermont-Ferrand, Les Cézeaux, Aubière Cedex BP265-63175, France; E-Mail: yuri.lapusta@ifma.fr

**Keywords:** granular flow, rheology, friction, spin, energetics, bifurcation

## Abstract

The flow of a dry granular material composed of spherical particles along a rotating boundary has been studied by the discrete element method (DEM). This type of flow is used, among others, as a process to spread particles. The flow consists of several phases. A compression phase along the rotating wall is followed by an elongation of the flow along the same boundary. Eventually, the particles slide or roll independently along the boundary. We show that the main motion of the flow can be characterized by a complex deformation rate of traction/compression and shear. We define numerically an effective friction coefficient of the flow on the scale of the continuum and show a strong decrease of this effective friction beyond a certain critical friction coefficient μ*. We correlate this phenomenon with the apparition of a new transition from a sliding regime to a rolling without sliding regime that we called the rolling transition; this dynamic transition is controlled by the value of the friction coefficient between the particle and the wall. We show that the spherical shape for the particles may represent an optimum for the flow in terms of energetic.

## 1. Introduction

Granular flows have received a lot of attention from physicists and mechanical engineers. Indeed, granular materials are ubiquitous in many industrial applications such as chemical engineering, mining and geosciences and unexpected behaviors can occur compared to classical fluids as jamming in silos (see for instance recently [1]). To date, we still have only a partial understanding of the basic principles the come to play such as the flow rules for such complex fluids: a unified description of their rheology from fundamental principles still poses great challenges [2,3].

Due to the various contact interactions between the constitutive particles of the flow (collisions and friction), several different length and time scales can be present which contribute to the complex behaviour of granular materials from a general point of view. Binary collisional interactions for dilute flows allow for a description in terms of a granular gas while, for denser flows involving frictional and multi-body contacts, additional transfers of elastic energy can occur through the network of the contacts between the beads.

A viscoplastic rheology has been highlighted recently for the description of such regimes with notable success when applied to different configurations of granular flow under shear [4] and this kind of approach has been extended recently to a flow in a 3D geometry [5].

However, from a general view point, we can wonder if internal degrees of freedom of the particles are always captured in such a frame at the macroscopic scale. For instance, interactions between particles under shear may also induce rotation and spin on these particles and this may also influence the rheology at a certain stage. 

In different cases of practical significance, granular flows may indeed be driven by forces other than gravity in which case the corresponding physical features may have to be revisited. Granular flows in centrifugal spreading are such an example.

**Figure 1:**
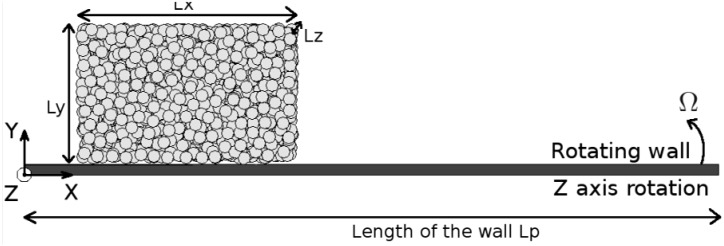
Schematic view of the flow of an assembly of grains along a rotating plane.

The flow in the monoparticle case has been studied previously both experimentally and theoretically [6,7,8]. The complexity of the frictional interaction and the energy dissipated during the interaction process between the bead and the rotating boundary was clearly brought to the fore. We showed indeed the relevance of defining two distinct friction coefficients *impact* m and *contact* m related to an impact and an enduring contact for a particle in motion along a fixed boundary. These informations will be useful for an optimization of the motion of a granule along a boundary (see for instance [9] in a context of terrestrial locomotion). Here, we study the flow of an assembly of particles along a rotating boundary through numerical simulations (velocity W, vertical axis of rotation (Z), see Figure 1). This situation is directly inspired by the process of centrifugal spreading of particles which is currently used in agricultural engineering.

Regarding the specific case of centrifugal spreading, previous studies were able to reproduce experimental trends qualitatively [10,11]. However, a quantitative agreement is still lacking partly because of the uncertainties regarding complex friction laws. The goal of the present paper here is different. As the energy in a granular flow is mainly dissipated through contacts between the particles by friction, a study of the flow on the scale of the contacts would be indeed of much value. In this letter, we present, from a physical viewpoint, some dynamical properties on the scale of the grains that appear numerically for these kinds of granular flows and that have an effect on the macroscopic scale. This information will be useful for an optimised control of the flow of the particles in terms of energetics whereas granular physicists have been more concerned until now in a geometric optimization of packings [12].

## 2. The Model

The granular flow consists of an assembly of N = 1500 particles of density r = 1768 kg/m^3^, radius *R* = 3 mm, mass *m* = 0.2 g and spring coefficient *K_n_* = 75 kN/m. The beads have no cohesion and no interstitial fluid is present between the grains so that the particles interact only via classical mechanical contact forces. In the initial configuration, the particles are set randomly in a rectangular box at the entrance of the vane (see Figure 1). The typical dimension of the box is equal to *L_x_* = 33 R, *L_y_* = 23 R and *L_z_* = 26 R. The length of the vane is equal to *L_p_* = 330 R.

We considered the case of a very dissipative material in order to be close to the situation of real particles and therefore chose a very low restitution coefficient *r* = 0.2. The numerical code is based on the Discrete Element Method (D.E.M.). This numerical approach is now classical in the field of granular physics and has been found to be useful in order to explore the properties of granular matter on the grain scale. The force model is chosen as the classical spring-dashpot model, *i.e.*, an elastic response with a viscous damping term in the force law.

For the sake of simplicity, we have modelled the interaction following the widely used approach introduced by P.A. Cundall and O.D.L. Strack [13]. This corresponds to a linear viscoelastic interaction between the particle and the vane modelled by a linear spring in parallel with a dashpot for the normal component (N). The tangential component (T) is modelled by a spring with Coulomb friction limit as follows:

(1) $$\begin{array}{l} N=K_{n}\delta_{n}-b_{n}\overset{•}{\delta_{n}}\\ T_{t+\Delta t}=-\text{min}({ℜ}_{t\to t+\Delta t}T_{t}+K_{t}\Delta \delta_{t},\mu \left|N_{t+\Delta t}\right|) \end{array}$$

*K_n_*, *K_t_* and *b_n_* are respectively the contact stiffnesses and the damping parameter: $K_{t}=\frac{2(1-\nu )}{\left(2-\nu \right)}K_{n}$ (Vu-Quoc *et al.* 2000 [14]) with the Poisson ratio ν = 0.3 and $b_{n}=2\text{ln}r\sqrt{mK_{n}}/\sqrt{\pi^{2}+\text{ln}r^{2}}$ (see [15]). *δ_n_* is the normal contact displacement also referred to as the overlap of the contacting bodies. The increment of tangential contact displacement $\Delta \delta_{t}={\overset{•}{\delta }}_{t}\Delta t$ is found by integrating the projection on the contact plane of the relative contact velocity. ${ℜ}_{t\to t+\Delta t}$ is the rotation matrix of the contact wall between time step *t* to t+Δt.

## 3. Physical Parameters of the Problem

The mechanical parameters which define this interaction law are:

- The dynamic friction coefficient between particles m;
- The dynamic friction coefficient between the particle and the wall *μ’*.

The coefficient of normal restitution r between two beads; this coefficient is linked through the spring-dashpot model by two parameters: the spring constant *n K* and the visco-elastic constant *b_n_*.

### 3.1. Collective Motion for the Particles

The combined inertial forces on the flow (centrifugal and Coriolis) induce four different stages for the flow: (i) an impact of the granular material along the boundary; (ii) a radial stretching of the flow along the boundary subjected to the centrifugal force; (iii) an ortho-radial compression of the granular material along the boundary induced by the Coriolis force; (iv) finally, an independent motion of the particles sliding or rolling along the boundary.

**Figure 2:**
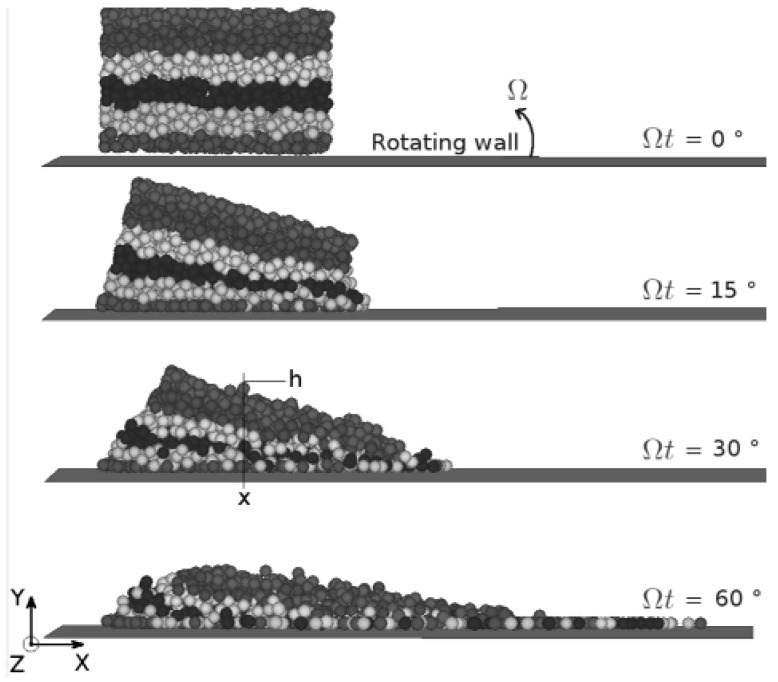
Schematic view of the flow at four angular displacements.

In order to study the collective motions of the beads, we numerically tracked the individual displacements of some of them in the bulk of the flow. A sketch of the evolution of the flow along the boundary at three different times is displayed in Figure 2. In that figure, we split the packing into several horizontal slices represented by different colors in grey scales for illustration purposes. The main phenomenon that can be observed on the picture is that the relative motion of the beads does not correspond to the classical shear motion between two adjacent layers of beads. On the contrary, we observe that two typical neighbouring beads move away from each other and let a bead from the above layer slip between them. We refer to this specific motion with the term traction-compression instead of shear. In the following paragraph, we quantify these different motions by introducing some non dimensional physical numbers which are characteristic of the physics of the flow.

### 3.2. Typical Physical Non Dimensional Numbers of the Flow

The inertial number *I* [4] is a non dimensional number which expresses the competition between the typical shear time scale (*T_γ_*) and the confinement pressure time scale (*T_c_*). This number is relevant to the classification of the different regimes of sheared flows [4]. Recently, a 3D visco-plastic formulation of this rheology was proposed. This is a Mohr-Coulomb like formulation with a friction coefficient dependent on the inertial number I [5]. Let us evaluate the local inertial number I defined in Equation 2:

(2) $$I=T_{c}/T_{\gamma }=\partial V_{x}/\partial yR\sqrt{\pi }/\sqrt{3\Omega \bar{V_{x}(h-y)}}$$

which is the ratio between the characteristic time of confinement *c T* by the Coriolis stress *P*, *i.e.*,

(3) $$T_{c}=\sqrt{m}/2RP=2R\sqrt{\pi \rho /6P}$$

and the shear time scale:

(4) $$T_{\gamma }=1/\overset{•}{\gamma }$$

where the shear rate is given $\overset{•}{\gamma }=\partial V_{x}/\partial Vy$, *V_x_* being the radial velocity of the flow. The coriolis stress *P* depends on the height (*h* − *y*) in the pile with h is the maximal height of the flow in (*Oy*) direction for a given x (see Figure 2). *P* is given by:

(5) $$P=2\rho \Omega \bar{V_{x}}(h-y)$$

with $\bar{V_{x}}=d\bar{x}/dt$ being the average radial velocity of the flow.

**Figure 3:**
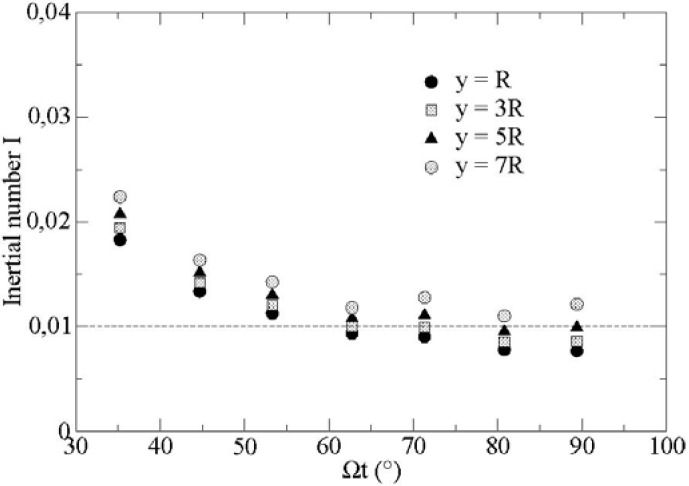
Inertial number *I* as a function of angular displacement (*x* = 33 R, *t* = 0.015 s, t = 0.015 s, *μ* = 0.1, *μ’* = 0.2; *y* = *R* – 3 *R* – 5 *R* – 7 *R*).

The present flow is somewhat non-standard because the shear rate $\partial V_{x}/\partial y$ is low compared to the traction rate $\partial V_{x}/\partial x.$ For instance, for values of Ω over 600 rpm, the ratio of the two gradients is above 5. The inertial number *I* decreases as a function of time and converges towards a value around *I* ≈ 0.01 (see Figure 3).

Furthermore, the classical inertial number *I* is not, in this case, the main characteristic parameter of the flow.

We can propose the introduction of new inertial numbers *J* and *J*’ defined by Equations 6 below in a similar way as it has been done in [4]. The inertial number *J* is the ratio between the characteristic time scale of confinement and the time scale of traction arising from the centrifugal stress in the (Ox) direction. Likewise, *J’* is the ratio between the characteristic time scale of traction of the centrifugal stress and the time scale linked to the compression in the (Oy) direction.

(6) $$\begin{array}{l} J=\partial V_{y}/\partial yR\sqrt{\pi }/\sqrt{3\Omega^{2}\bar{x}(h-y)}\\ J'=\partial V_{x}/\partial xR\sqrt{\pi }/\sqrt{3\Omega \bar{V_{x}}(h-y)} \end{array}$$

**Figure 4:**
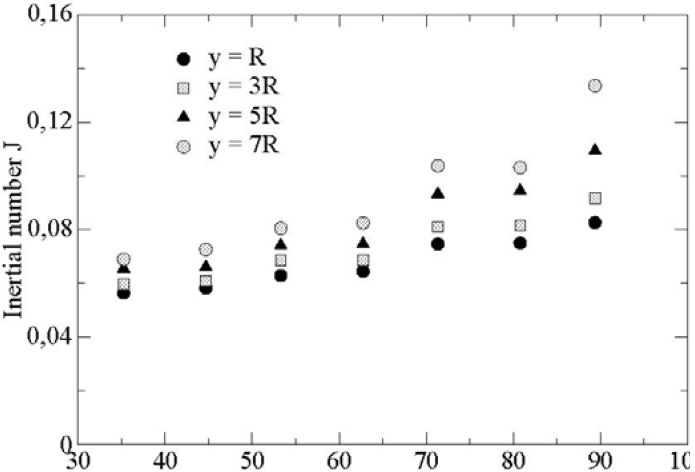
Inertial number J as a function of angular displacement (*x* = 33 R, *t* = 0.015 s, *μ* = 0.1, *μ’* = 0.2, *y* = *R* – 3 *R* – 5 *R* – 7 *R*).

We display the inertial number *J* on Figure 4. The ratio between the two inertial numbers does not vary significatively along the direction (*Oy*) for the range of parameters considered. 

## 4. Chararacterization of the Friction of the Flowing Granular Material along the Rotating Boundary

We probe the flow by introducing a rectangular box which is fixed with respect to the boundary and in which some physical properties on the scale of the continuum at the interface can be calculated. The basis of this box has a side length equal to 10 bead diameters. This has been found to be sufficient to describe the local properties for the flow and at the same time avoid any “grain scale” effect linked to the microstructuration in the flow. The height of the box is chosen to be 1/10 of a radius R in order to detect only the contacts between the beads and the boundary. This box is positioned in contact with the vane at a distance *x* = 50 *R* in our specific case.

We define for this purpose an effective friction coefficient of the granular material flowing along the vane *μ_eff_* in this box as the ratio between the sum of tangential shear forces at the contact Σ*T* to the sum of the normal forces Σ*N*, namely:

*μ_eff_* = Σ*T/*Σ*N* (7)

**Figure 5:**
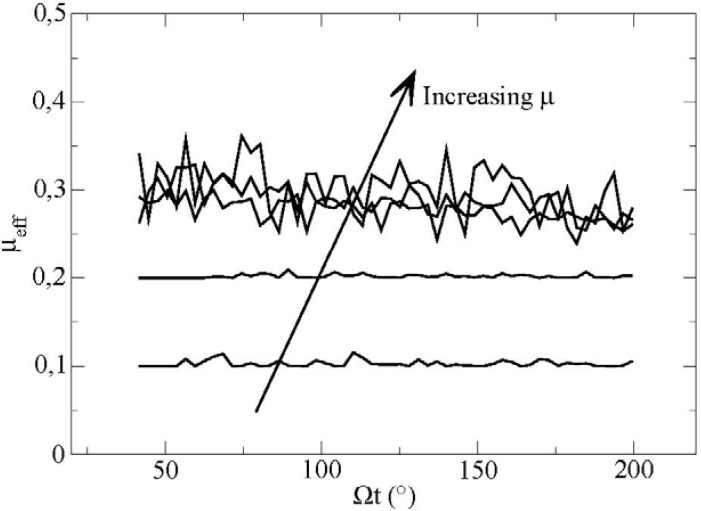
Effective friction at the boundary as a function of time for different friction coefficients particle/vane measured in the elementary volume for values of the microscopical parameters **(***μ’* = 0.2 and m = 0.1−0.2−0.3−0.4−0.5).

The sum of both forces is made on all the contacts between the boundary and the particles inside the box. The evolution of *μ_eff_* as a function of time for different values of the friction coefficient m is plotted in Figure 5.

We observe first positive fluctuations of the mean friction coefficient. These fluctuations are very weak for values of m which are smaller or equal to 0.2. For higher values of the friction coefficient, we observe that the fluctuations become much more important, as much as 30% in relative value. 

We can ask ourselves about the physical origin of these strong fluctuations on the value of the effective friction coefficient. For this purpose, we tracked the motion of individual particles along the boundary with respect to velocity and spin.

**Figure 6:**
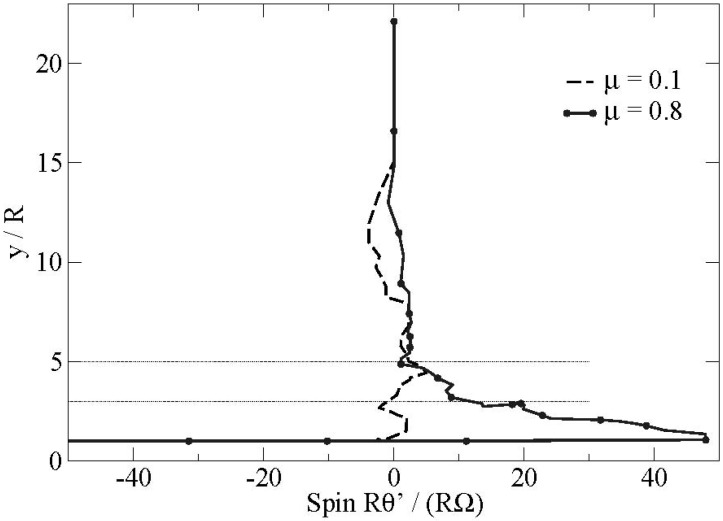
Evolution of the angular velocity of a particle in the flow for different friction coefficients at the boundary (μ = 0.1–0.8).

**Figure 7:**
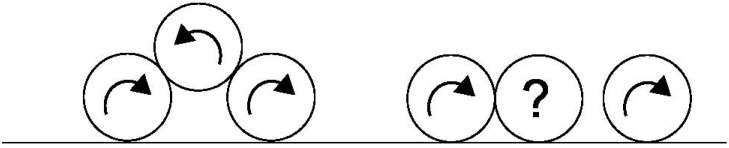
Schematization of the frustration in the rotations of the particles in the first layers of the flow.

We present in Figure 6 the evolution of the angular velocity of a particle $R\overset{•}{\theta }$ in the flow for two extreme values of the friction coefficient at the boundary: μ = 0.1 and μ = 0.8. We also bserve that the particles changing layers with high friction coefficient have a spin in the opposite direction $R\overset{•}{\theta }$ > 0 to the spin of the particle in the layer below or near the boundary $R\overset{•}{\theta }$ < 0. Additionally the contact of the bead on the boundary is associated with a phenomenon of strong frustration of the rotations for the particles with high friction coefficient (see Figure 7). This phenomenon is localized in the first layers of the flow. This frustration of the rotations induces naturally an additional friction along the boundary during a certain transitory. This scenario represents a strongly plausible explanation for the presence of these fluctuations.

**Figure 8:**
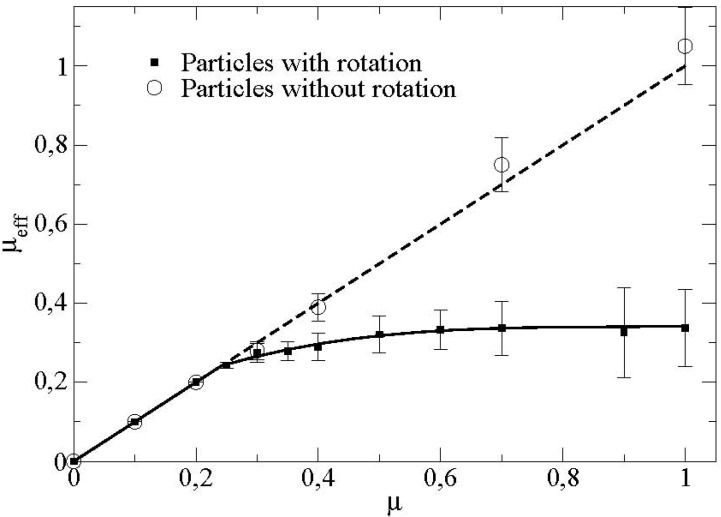
Mean effective friction at the boundary as a function of the friction coefficient particle/vane for particles with and without rotational degrees of freedom (*μ’* = 0.2).

Figure 8 displays furthermore the dependence of this effective friction coefficient with respect to the friction particle/wall.

We observe a linear increase of the friction coefficient as a function of the microscopic basal friction. The slope of the curve is equal to 1. This shows that the effective friction corresponds to the microscopic friction particle/wall. Above a certain critical friction coefficient (around μ* ≈ 0.25 in this case), we observe a saturation of the effective friction coefficient towards a value here around 0.3. We observe in particular that this value of the critical friction coefficient at the transition μ* is different from the value of the critical friction coefficient for the apparition of the rolling without sliding regime in the case of a single particle flowing in the same rotating device [5].

For the same flow configuration, we present also in Figure 8, the effective friction when we blocked the rotations of the particles. We showed that the effective friction coefficient does not decrease anymore but remains equal to the microscopic friction coefficient particle/boundary. This shows that the transition observed is directly connected to the rotations of the particles in the flow. In order to gain more insight into these phenomena, we introduced the sliding ratio *R_s_* and the rolling ratio *R_t_* at the boundary where these two quantities are linked by the simple relation *R_t_* = 1 − *R*.

We define the sliding ratio *s R* for the *i N* particles in contact with the boundary in the elementary volume as:

(8) $$R_{s}=1/N{|}_{u}\sum_{i=1}^{i=N_{i}}\left[\overset{•}{x_{i}}-R\left|\overset{•}{\theta_{i}}\right|\right]/\overset{•}{x_{i}}$$

**Figure 9:**
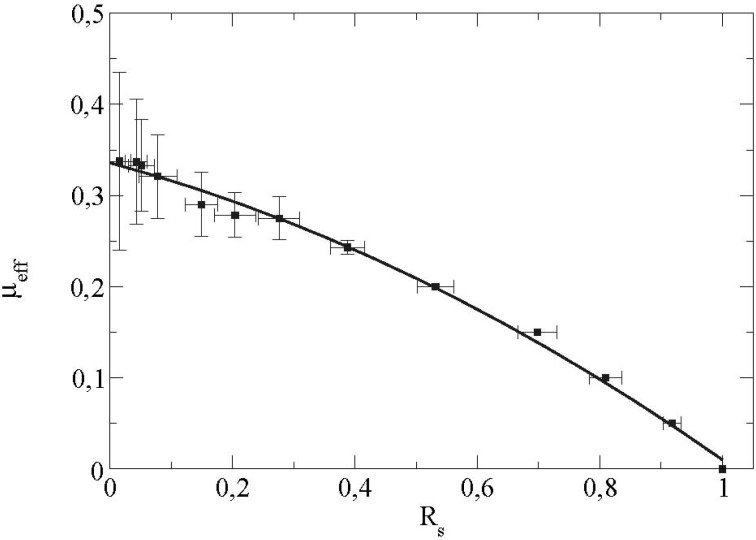
Effective friction at the boundary as a function of the sliding ratio *R_s_* *=* 0.2.

As the spin of the particles is created by the friction forces, the rolling ratio *t R* increases with the microscopic friction coefficient (particle/wall). The evolution of the effective friction with R is sketched on Figure 9.

**Figure 10:**
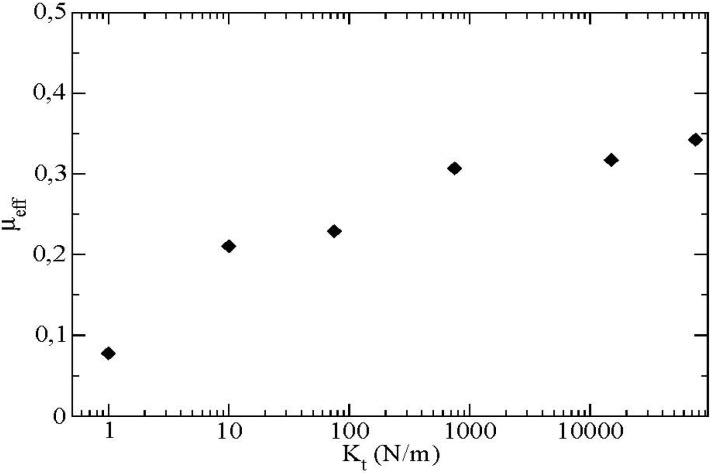
Effective friction at the boundary as a function of the tangential contact stiffness *Kt*, *μ* = 0.4, *μ’* = 0.2.

We see that as *μ_eff_* increases, the value of the sliding ratio *s R* decreases. The effective friction coefficient is saturated at a value equal to 0.33 for a sliding ratio equal to 0 (*i.e.*, a rolling without sliding state). This corresponds indeed to the value of the saturation observed on the previous curve (Figure 8). This saturated value of the effective friction coefficient depends essentially on certain microscopic parameters of the contact force law such as the tangential contact stiffness *Kt* as displayed in Figure 10.

## 5. Mobilization of the Sliding/Rolling Contacts in the Bulk of the Flow

**Figure 11:**
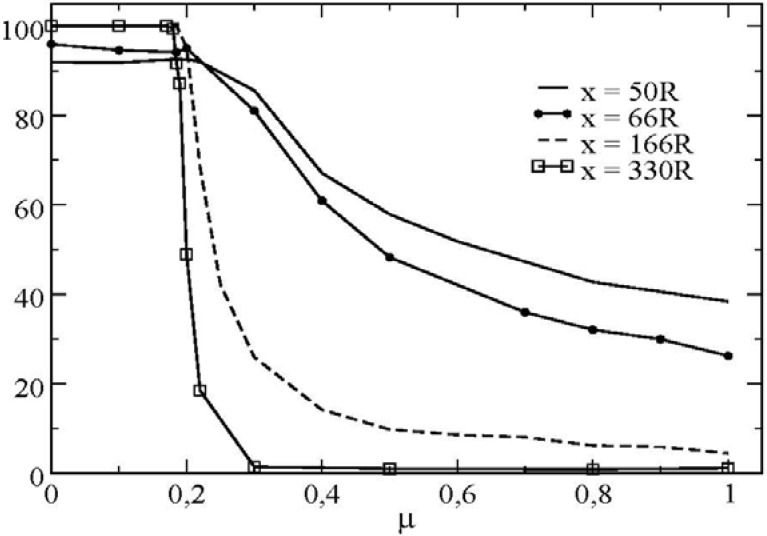
Proportion of sliding contacts cumulated in time as a function of the friction coefficient (Ω = 500 *rpm*; *μ’* = 0.4).

We can now illustrate, for one set of microscopical parameters, the influence of the sliding/rolling contacts between particles in the bulk of the flow. Figure 11 represents the proportion of sliding contacts as a function of the friction coefficient particle/wall. The figure demonstrates again the signature of the transition from a sliding regime towards a rolling without sliding regime; this transition is triggered by the value of the friction coefficient at the wall. We observe a strong decrease in the ratio of sliding contacts in the bulk of the flow above a certain critical friction coefficient. We see also that the transition curve is not invariant along the radial direction.

## 6. Energetical Considerations in the Granular Flow

In this section we would like to characterize the flow from an energetic point of view. The energy communicated by the rotation of the vane is partitioned between translational and rotational kinetic energy, frictional energy and at finally collisional energy.

The different quantities are defined below:

  - Kinetic energy of translation *E_t_* and rotation *E_r_*:

(9) $$E_{t}+E_{r}=1/2\sum_{i=1}^{i=N}m{\left(\overset{•}{x_{i}}\right)}^{2}+I{\left(\overset{•}{\theta_{i}}\right)}^{2}$$

  - Frictional energy *E_f_*:

(10) $$E_{f}=\sum_{t}\sum_{i=1}^{i=c}m(T_{i}^{t})\Delta U_{i}^{t}$$

where $T_{i}^{t}$ is the friction force at the contact and at the considered time step and $\Delta U_{i}^{t}$ is the increment of tangential displacement during the considered time step.

  - Elastic energy of deformation *E_d_*:

(11) $$E_{d}=1/2\sum_{i=1}^{i=c}N_{i}^{2}/K_{n}+T_{i}^{2}/K_{t}$$

where *c* corresponds to the number of contacts between particles in the flow. This energy is found to be proportionally low compared to the kinetic and frictional energy [8].

**Figure 12:**
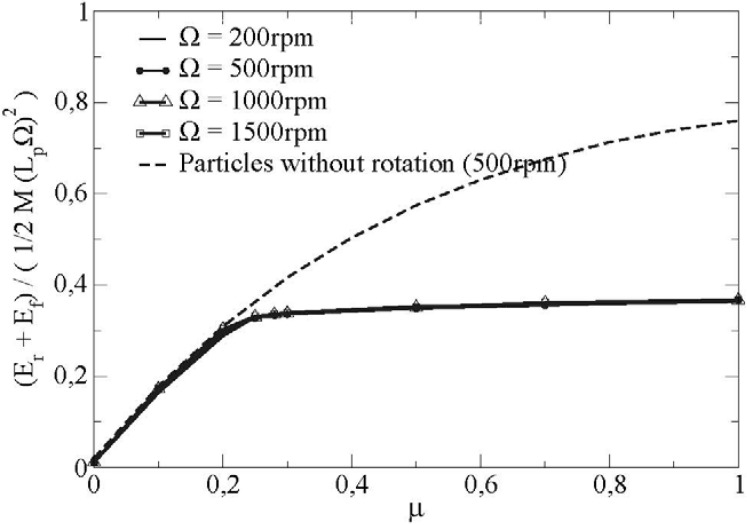
Comparison of the sum of the energy dissipated by friction *E_f_* and the kinetic energy of rotation *E_r_* at a distance *x* = 166 *R* for particles with and without rotational degrees of freedom (variation of Ω, *μ’*= 0.4).

The evolution of the sum of the energy dissipated by friction and the kinetic energy of rotation is represented on figure Figure 12 for one set of microscopical parameter and different rotating velocities of the vane.

In this figure, we observe also the transition from a sliding regime towards a rolling without sliding regime. We note a saturation of the sum of the energy from a critical value of the friction coefficient, this satured value being independent of the rotational velocity of the vane.

In the same way, we show the sum of the energy for particles with blocked rotations. In this case, the rotational energy is zero. Beyond the critical friction coefficient, the frictional energy increases because all the contacts are sliding. Furthermore, Figure 12 tends to show that the transition from a sliding regime towards a rolling without sliding regime reduces the dissipated energy in the bulk of the flow.

## 7. Discussion

The reduction of the effective friction coefficient along a boundary induced by the presence of rolling contacts between beads has been also observed in other different systems. For instance, the case of a confined bidimensional packing of cylinders under quasistatic shear [16]. This effect has also been identified previously in numerical simulations of rapid collisional granular flows in a couette geometry (see [17]). From a general point of view, the dependence of the effective friction coefficient with respect to the micromechanical properties of the spinning beads is still unclear notably in experiments where the dissipation of the energy at the scale of the contact is poorly known. This may have a crucial influence on the dynamics and represents also important issues for applications [18].

## 8. Conclusions

The simulation results presented in this article bring to the fore some new properties for a granular material flowing along a rotating wall, on a qualitative stage. In this device, the material is stressed in traction in the radial direction and in compression in the ortho-radial direction from the vane as well as sheared because of the presence of the wall.

We were specifically interested in the friction of the granular flow along the rotating boundary. Most interestingly, we find that the effective friction coefficient at the scale of the continuum is on average constant and equal to the microscopical friction coefficient for low friction coefficients particle/wall.

Also, it decreases strongly when the friction particle/wall exceeds a certain microscopic critical friction coefficient μ*. This behavior is directly linked to the apparition of a transition towards a rolling without sliding regime for the flowing particles at the boundary.

We stress the fact that this kind of transition from sliding to rolling without sliding is a new feature in the physics of dense frictional granular flows. The dependance of the critical friction coefficient μ* remains now to be clarified. A more precise analytical characterization of this rolling transition would be also highly relevant to carry out in the near future.

We gave also some first numerical indications that this transition has a direct influence on the limitation of the dissipated energy in the flow for particles of spherical shape. If such a transition can be reached for real particles, it may present in fact a practical interest with regard to a reduction of the dissipated energy in the flow.
